# Abnormalities of early folliculogenesis and serum anti-Müllerian hormone in chinese patients with polycystic ovary syndrome

**DOI:** 10.1186/s13048-021-00786-0

**Published:** 2021-02-18

**Authors:** Juan Du, Xiangyan Ruan, Fengyu Jin, Yanglu Li, Jiaojiao Cheng, Muqing Gu, Alfred Otto Mueck

**Affiliations:** 1 Department of Gynecological Endocrinology, Beijing Obstetrics and Gynecology Hospital, Capital Medical University, No. 251, Yaojiayuan Road, Chaoyang District, 100026 Beijing, People’s Republic of China; 2 Research Centre for Women’s Health and University Women’s Hospital of Tuebingen, University of Tuebingen, Tuebingen, Germany

**Keywords:** PCOS, Early folliculogenesis, Follicle density, AMH

## Abstract

**Purpose:**

To investigate abnormalities of early folliculogenesis and Anti-Müllerian hormone (AMH) concentrations in polycystic ovary syndrome (PCOS) patients, and to analyze the association between AMH and early-stage follicle densities (FD).

**Methods:**

A total of 175 patients underwent ovarian tissue cryopreservation in the first official cryobank in China, of which 16 patients aged 30–40 years old were diagnosed with endometrial cancer (all without initial chemo/radiotherapy), including 5 patients with concurrent PCOS and the other 11 patients without. We obtained standard cortical biopsies to measure FD using calcein staining. Blood samples were collected before cryopreservation to evaluate AMH concentrations.

**Results:**

PCOS showed nearly three times the primordial and primary FD than NPCOS (*P* = 0.027), as well as more secondary preantral follicles (*P* = 0.002). A significantly higher proportion of secondary preantral follicles and a lower proportion of primordial and primary follicles were observed in PCOS (*P* = 0.01). Furthermore, the AMH concentration in PCOS was four times higher than that in NPCOS (*P* = 0.003), which is significantly correlated with primordial and primary follicle densities (*r* = 0.855, *P* < 0.001) and secondary preantral follicle densities (*r* = 0.732, *P* = 0.007).

**Conclusions:**

We found significant disorders of early folliculogenesis in PCOS, which showed close correlation with increased AMH concentrations. To our knowledge, abnormalities of early-stage follicles have been shown for the first time in ovarian tissue of Chinese PCOS women. We suppose that the elevated AMH level is associated with abnormalities of early folliculogenesis within the complex PCOS pathogenesis, which may explain why AMH has the potential to be used as a biomarker for the diagnosis of PCOS. Our findings provide more implications for understanding the mechanism of PCOS, and new directions for further studies.

## Background

Polycystic ovary syndrome (PCOS), the most common endocrinopathy in reproductive age, affects 8–13 % of women worldwide [1]. PCOS is characterized by ovulatory dysfunction, hyperandrogenism and polycystic ovarian morphology, which is also associated with an increased risk of infertility, type 2 diabetes, obstetrical complications and endometrial carcinoma [2]. The underlying mechanism of PCOS is complex and remains uncertain [3]. Genetics has been thought to play an essential role in the pathogenesis of PCOS [4]; to date, almost 100 genes related to PCOS have been identified by a large number of studies [2]. For this reason, dependence of the pathogenesis on different ethnicities has been discussed [5]. Although a great proportion of studies on PCOS have been performed in the Western world, a high prevalence of PCOS is noticeable in China. For example, according to a large community-based study with 15,924 eligible participants, the prevalence of PCOS was 5.6 % [6].

In anovulatory PCOS, the later stages of follicular development have been proved to be abnormal [7], whereby the arrested growth of small antral follicles is one of the most distinctive characteristics of PCOS [8, 9]. However, few studies have focused on earlier and even primordial follicular stages in PCOS ovarian tissue and, to our knowledge, until now no study at all has been published about this for Chinese women.

Anti-Müllerian hormone (AMH) is mainly secreted by the granulosa cells of large preantral and small antral follicles [10–12]. AMH levels are low in prepubertal girls, rise during early puberty and reach a maximum at the average age of 20–25 years, followed by a gradual decline to a minimum around menopause [3, 9]. Higher levels of AMH are found to be associated with PCOS [2, 3, 12–15]. However, it is unclear if AMH is only a biomarker of PCOS or plays an essential role in the PCOS pathogenesis [16]. Furthermore, it is well known that AMH is associated with the more massive amount of small antral follicles in PCOS. Nevertheless, the association between AMH and the early stages of follicles has rarely been studied.

We previously published studies about the possible value of AMH measurements in PCOS patients, especially for the assessment of disease severity in the different phenotypes [14, 15]. In our present study, we assessed AMH to investigate if there could be an association with early folliculogenesis of PCOS. Since we were able to successfully demonstrate the good quality of human ovarian tissue after storage using international “SOP guidelines” in our new “International Ovarian Tissue Cryobank”, which is the first, and to our knowledge, still the only one in China [17], we decided to perform a study using ovarian tissue from this bank. We aimed to focus on the early stages of follicle development, comparing the density and proportion of follicles in ovarian tissue between PCOS and non-PCOS patients, and analyze the correlation between AMH serum concentrations and follicle densities in PCOS.

## Materials and methods

### Patients

**Fig. 1:**
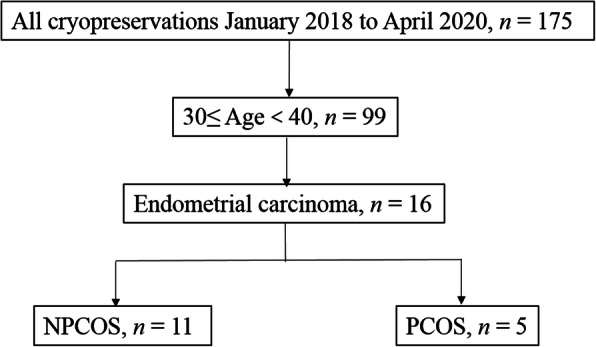
Recruitment of cryopreserved endometrial carcinoma patients combined with PCOS or without

The current study was performed at the first official “International Ovarian Tissue Cryobank” in China, Beijing Obstetrics and Gynecology Hospital, Capital Medical University, Beijing, China, and approved by the hospital ethics committee (NO. 2017-KY-020-01). A total of 175 patients underwent ovarian tissue cryopreservation before gonadotoxic treatments between January 2018 and April 2020. Among these cancer patients cryopreserved in our cryobank, only patients with endometrial cancer had PCOS, and there was no history of PCOS in other patients. PCOS patients were diagnosed according to Rotterdam criteria, who had two of the following three criteria: (1) Oligo- and/or anovulation; (2) Polycystic ovarian morphology (PCOM); (3) Clinical and/or biochemical signs of hyperandrogenism [18]. The patients were aged between 30 and 40 years (*n* = 99) and 16 were diagnosed with endometrial carcinoma. Five of these endometrial carcinoma patients had concurrent PCOS (PCOS group) and the other 11 patients did not (NPCOS group) (Fig. 1). Informed consent was obtained from all participants.

For each patient, ovarian tissue was removed by laparoscopy and was transported to our cryobank in sterile Custodiol medium (Dr. Franz Köhler Chemie, Bensheim, Germany) at a temperature of 4 ~ 8℃. Cortical fragments (8 × 4 × 1 mm) were prepared for cryopreservation; meanwhile, standard biopsies were obtained using punches (PFM Medical AG, Cologne, Germany) for follicle density analysis and routine viability assay.

### Analysis of follicle density

To evaluate the follicle density (FD) and to reduce heterogeneity, two 3mm biopsies (January 2018 to August 2018) or four 2mm biopsies (September 2018 to April 2020) from different cortex areas were collected for each patient [10]. Cortical biopsies were then incubated in a 5 % CO2 incubator at 37℃ for 2 hours in 0.8 mg/ml collagenase Type 1A (Sigma-Aldrich Chemie GmbH, Munich, Germany) supplemented with 0.2 % calcein-AM (Promega GmbH, Mannheim, Germany) and DPBS (Gibco by Life Technologies, Paisley, UK) for digestion and staining. After pipetting and placing at room temperature for 30 minutes, follicles descended to the bottom of the well so that FD could be measured by fluorescence and light microscopy. Primordial or primary follicles were identified by a single layer of flattened or cuboidal granulosa cells (GCs) [19]. Secondary preantral follicles were identified by two or more layers of GCs, as well as the absence of antrum [20]. The meander-shaped counting method was used to determine the number of follicles in each well [10]. To reduce the bias, follicles were blindly counted by two independent observers. Then we calculated the FD for every patient to make values comparable, converting the follicle numbers in 2 × 3mm or 4 × 2mm biopsies to the volume of one 3mm biopsy.

### Analysis of AMH

Before laparoscopy and cryopreservation, we collected blood samples from patients to evaluate their serum AMH levels. According to the manufacturer’s instruction, blood samples were centrifuged and measured in our clinical laboratory directly after collection using AMH ELISA Kits (Kangrun, Guangzhou, China). The intra-assay and inter-assay coefficient of variation was less than 5 % and 10 %, respectively. The detection range of AMH was 0.24–11.78 ng/ml.

### Statistical analyses

Statistical analyses were carried out by SPSS 16.0 (IBM corporation, New York, USA). The age, Body Mass Index (BMI), transportation temperature, and serum AMH levels were presented as the mean ± standard deviation (SD), whose mean values between the two groups were compared by using the *t*-test. Follicle counts were presented as the median and interquartile range (IQR), and the independent sample Mann-Whitney test was used to compare results. Differences between ratios were tested by Chi-Square tests. Relationships between follicle counts and AMH concentrations were evaluated by Spearman’s test to determine correlation coefficients. The differences with *P *< 0.05 were considered to be significant.

## Results

### Characteristics

In this study, the age of PCOS patients (34.6 ± 2.4 years, range 32–38 years) was similar to NPCOS patients (34.1 ± 2.1 years, range 31–39 years, *P* = 0.671). Most of the patients were obese, and BMI showed similarities between two groups (PCOS 31.1 ± 9.6 kg/m^2^, NPCOS 25.1 ± 5.6 kg/m^2^, *P* = 0.166). None of patients were smokers. There was also no statistically significant difference in the ovarian tissue transportation temperature between PCOS patients (5.9 ± 0.8 °C, range 4.6-6.9°C) and NPCOS patients (6.1 ± 1.4°C, range 4.4-8.0°C, *P* = 0.823). None of the patients in this study had received chemotherapy or radiotherapy before ovarian tissue removal and cryopreservation, and ovarian tissue biopsies were all superior in quality without coagulation. Detailed characteristics are shown in Table 1.

**Table 1:** Characteristics of patients with and without PCOS ovaries

Case number	Age (years)	BMI (kg/m^2^)	Smoking	Menstrual Cycle (days)	PCOM	Hyperandrogenism	Pregnancy history	Chemo/ radiotherapy before cryopreservation	Temperature of ovarian transportation (℃)	Follicle density / 3mm biopsy			AMH (ng/ml)
										**Total**	**Primordial and primary**	**Secondary pre-antral**	
**PCOS 1**	38	20.0	No	5–7/30–90	Yes	No	G0P0	No	6.9	27	23	4	12.18
**PCOS 2**	34	41.5	No	9/30–90	Yes	Yes	G0P0	No	4.6	74	68	6	9.26
**PCOS 3**	36	40.4	No	10–14/15–60	Yes	Yes	G0P0	No	6.0	16	14	2	6.95
**PCOS 4**	32	24.2	No	2–3/30–180	Yes	No	G0P0	No	6.0	90	72	18	13.94
**PCOS 5**	33	29.4	No	2–3/60–90	Yes	No	G1P1	No	6.0	18	15	3	2.64
**NPCOS 1**	31	29.0	No	5–7/28	No	No	G0P0	No	6.4	6	6	0	0.17
**NPCOS 2**	32	21.0	No	6–7/28	No	No	G0P0	No	4.4	33	32	1	4.27
**NPCOS 3**	34	N/A	No	3–5/60–90	No	No	G0P0	No	6.2	15	14	1	1.59
**NPCOS 4**	34	33.3	No	7/28	No	No	G0P0	No	7.4	3	3	0	N/A
**NPCOS 5**	34	19.1	No	8–9/28–30	No	No	G0P0	No	7.6	12	12	0	4.16
**NPCOS 6**	39	18.8	No	7/35	No	No	G1P0	No	4.5	11	9	2	0.29
**NPCOS 7**	33	20.2	No	3/40–45	No	No	G0P0	No	4.5	5	5	0	1.54
**NPCOS 8**	35	25.0	No	7/15–60	No	No	G0P0	No	5.6	18	17	1	N/A
**NPCOS 9**	35	N/A	No	7/30	No	No	G1P1	No	8.0	27	24	3	N/A
**NPCOS 10**	35	30.9	No	7/30	No	No	G0P0	No	4.7	2	2	0	0.57
**NPCOS 11**	33	28.9	No	5/30	No	No	G1P0	No	7.3	5	5	0	N/A

*N/A* Not available

### Follicle density

**Fig. 2:**
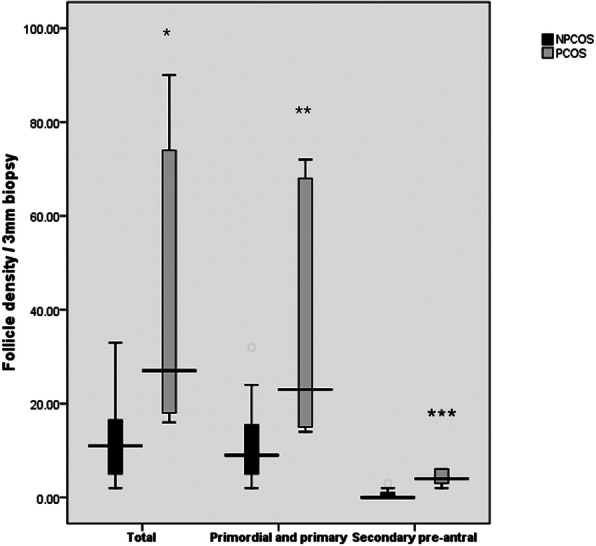
Box and whisker plots show comparison of total, primordial and primary, and secondary preantral follicle density per 3mm biopsy in NPCOS (black) and PCOS (grey), respectively. Horizontal lines represent median, boxes represent 75th and 25th percentiles, and whiskers represent internal limits. * *P* = 0.019, ** *P* = 0.027, *** *P* = 0.002

**Fig. 3:**
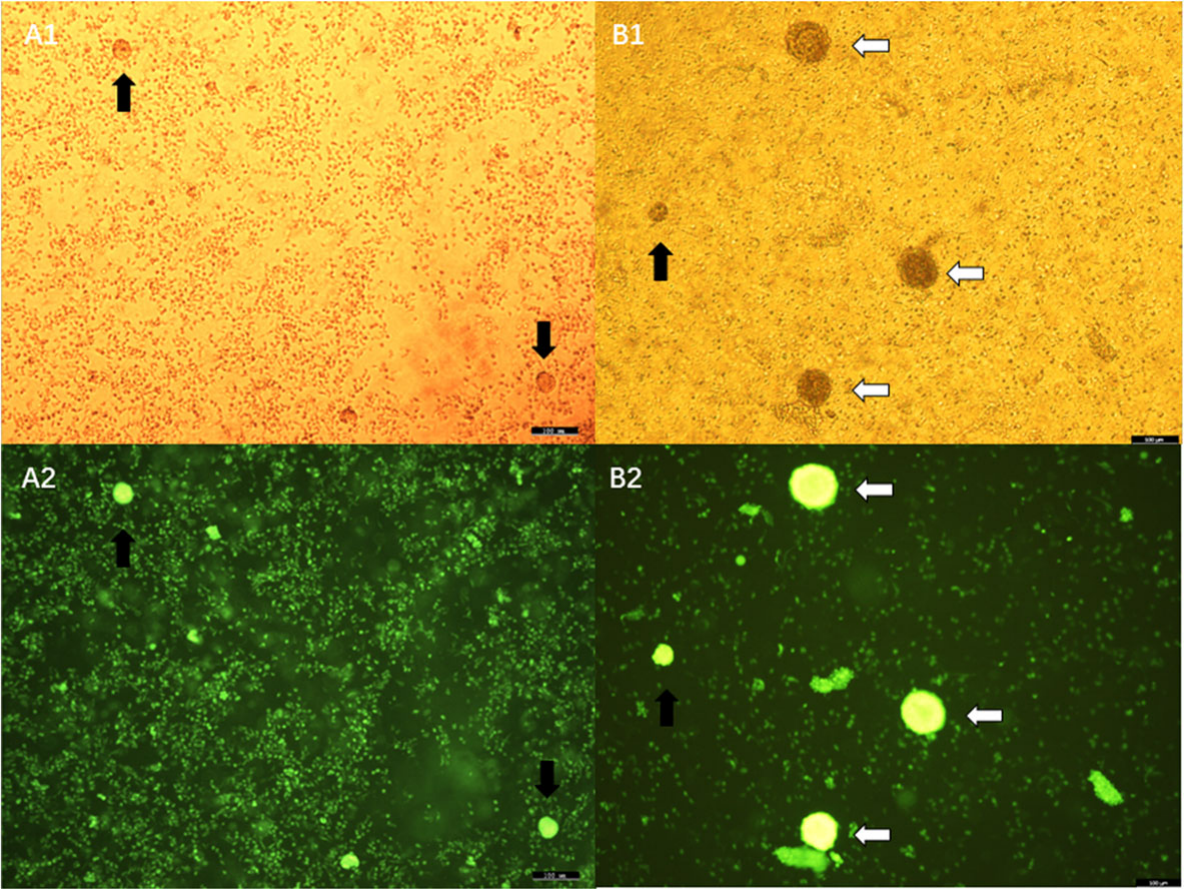
Follicles in cortical biopsies after digestion with collagenase and staining with calcein from NPCOS (A1 and A2) and PCOS (B1 and B2), observed by light (top row) and fluorescence light (bottom row). Black arrow, primordial or primary follicles; White arrow, secondary preantral follicles; Bar, 100µm

The number and composition of follicles in PCOS and NPCOS patients were analyzed and compared in this study. The total follicle number per 3mm biopsy in PCOS patients (median 27, IQR 17–82) was about three times that of NPCOS patients (median 11, IQR 5–18; *P* = 0.019). Of the whole follicle population, PCOS patients had nearly three times the primordial and primary follicles (median 23, IQR 14.5–70) than NPCOS patients (median 9, IQR 5–17; *P* = 0.027), and there were also significantly more secondary preantral follicles in PCOS patients (median 4, IQR 2.5–12) than that in NPCOS patients (median 0, IQR 0–1, *P* = 0.002) (Figs. 2 and 3).

In PCOS ovarian biopsies, primordial and primary follicles represented 85.3 % of the total follicle number, and secondary preantral follicles accounted for 14.7 % of the total. Of all the NPCOS follicles counted, 94.2 % were primordial and primary follicles, and only 5.8 % were secondary preantral follicles. When using the Chi-square tests to evaluate the differences in ratios, a significantly higher proportion of secondary preantral follicles and lower proportion of primordial and primary follicles were observed in PCOS patients than that in NPCOS patients (*P* = 0.01).

### Serum AMH level

**Fig. 4:**
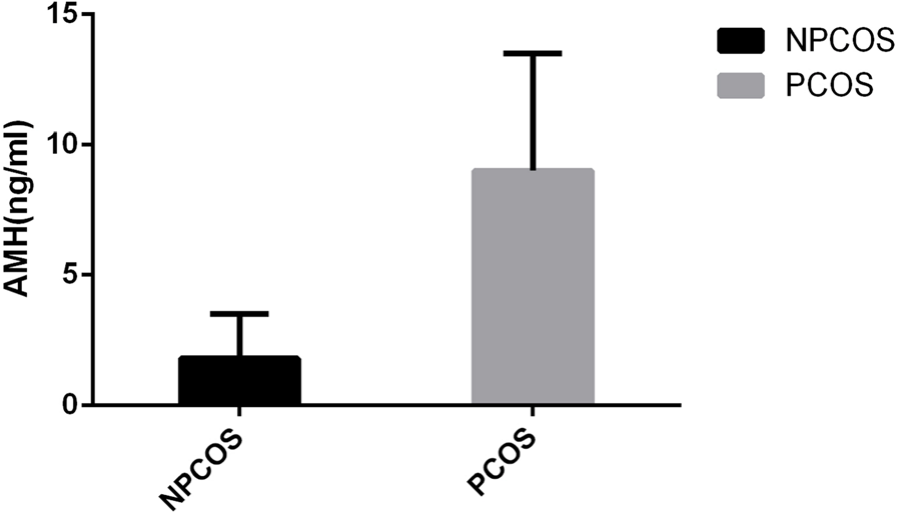
PCOS showed four times higher AMH concentrations when compared with NPCOS, *P* = 0.003

Serum AMH levels of all PCOS patients (*n* = 5) and most NPCOS patients (*n* = 7) were measured. Not surprisingly, PCOS patients with 9.0 ± 4.5 ng/ml (range 2.64–13.94 ng/ml) showed 4 times higher AMH concentrations when compared with NPCOS patients (1.8 ± 1.7 ng/ml, range 0.57–4.16 ng/ml, *P* = 0.003) (Fig. 4). Furthermore, the correlation analysis between AMH levels and follicle numbers was performed in these 12 patients having available AMH data. AMH levels are not only significantly correlated with secondary preantral follicle densities (*r* = 0.732, *P* = 0.007), but are also statistically associated with primordial and primary follicle densities (*r* = 0.855, *P* < 0.001).

## Discussion

In our study, a significantly lower proportion of small preantral follicles and a higher proportion of secondary follicles were observed in PCOS compared to NPCOS. According to previous studies, we defined primordial, intermediary and small primary follicles as resting follicles [10, 19, 21], because the extremely slow transformation from primordial follicles into primary follicles seems to be a maturation rather than a growing process [19]. Thus, this finding illustrates that a higher proportion of resting follicles in PCOS ovaries leave the initial pool and start growing, which is supported by other studies [7, 22].

With the increased initial follicular recruitment, PCOS would be expected to have a lower number of resting follicles. However, our current study found that not only the density of secondary preantral follicles is significantly increased in PCOS, but also the population of small preantral follicles, including primordial and primary follicles, exceeding that of NPCOS ovaries threefold. From these findings we suggest, as other authors have also suspected [5, 23], that the overall density of follicles in PCOS is increased, and the defects in growth may begin at the earliest stages of folliculogenesis in PCOS.

Possible explanations for the increased follicle density could be a higher initial follicle population and/or less atresia. Many factors can influence the primordial germ cells’ specification and migration, cell division, and primordial follicle formation [20], which may cause the abnormality of the primordial follicle pool in PCOS. On the other hand, it has been demonstrated that the rate of atresia throughout the culture period in follicles was significantly lower in PCOS tissue [24].

We found that the mean serum AMH level in PCOS patients was four times higher than that in NPCOS patients, and significant correlations were found between AMH levels and early-stage follicle densities. The explanation is obvious as higher AMH levels in PCOS may attribute to the more massive amount of small antral follicles in PCOS. If true, the question arises as to why AMH concentrations are closely correlated with early-stage follicles? We suppose that AMH is not only a biomarker of PCOS, but may also be an essential factor in the pathogenesis of PCOS.

Follicular growth is determined by the balance of inhibitory and stimulatory factors. The TGF-β family is considered to be the prime candidate for regulating follicle development [25], of which AMH is a crucial member. AMH has been proven to act as a vital inhibitor for follicular growth, including FSH-independent initial recruitment and FSH-dependent dominant selection [3, 12, 16, 26, 27]. However,in primordial and primary follicles from PCOS, AMH expression was found to be decreased [23], resulting in reduced AMH inhibition, enhanced follicular growth, and following hypersecretion of AMH. Besides, the hypothesis that AMH inhibits follicular atresia has also been put forward and verified by some in vitro and animal experiments [28]. Collectively, the disorder of AMH expression in early-stage follicles might allow for advanced follicular development, higher concentration of AMH, and less atresia of follicles, which can explain the higher proportion of growing follicles and the larger number of early-stage follicles observed in the current study.

However, the regulation of AMH production is still far from clear. The increased AMH production in PCOS patients may be related to elevated LH, androgen levels, and insulin resistance [12, 14, 16]. According to a very recently published hypothesis of Dozortsev et al. which can stimulate for further research on this issue, a higher cumulative level of progesterone following increased follicular-phase recruitment of follicles may also trigger PCOS [29]. On the other hand, what if the larger initial follicle pool is the foremost cause of PCOS? We suppose that for some reasons, the higher initial population of primordial follicles arises in the pool, leading to an increased number of growing follicles, producing the original higher concentration of AMH. To prove this hypothesis, further studies on ovarian tissue are needed.

## Strengths and limitations

This is the first study in which abnormalities of early-stage follicles in ovarian tissue and correlations between early folliculogenesis and serum AMH have been investigated in Chinese PCOS women. We maintained the strictly high homogeneity of this study. We recruited patients of comparable age, all with endometrial carcinoma and without chemo/radiotherapy before cryopreservation and the study was conducted in the only center using same procedures between two groups. One limitation of our study is that the patient sample seems to be low. However, a great number of follicles (total *n* = 380) from ovarian tissue have been analyzed, and the AMH difference between PCOS and NPCOS was very high with a remarkable significance (4-fold higher, *P* = 0.003).

## Conclusions

In the present study, we found significant disorders of early folliculogenesis in PCOS, which showed close correlation with increased AMH concentrations. We suppose that the elevated AMH level is not only a clinical manifestation caused by the increasing number of small antral follicles in PCOS, but is also associated with abnormalities of early folliculogenesis within the complex PCOS pathogenesis, which may explain why AMH has the potential to be used as a biomarker for the diagnosis of PCOS. Our findings provide more implications for understanding the mechanism of PCOS, and new directions for further studies.

## Data Availability

The datasets used and/or analyzed during the current study are available from the corresponding author on reasonable request.

## Abbreviations

AMH: Anti-Müllerian hormone
BMI: Body Mass Index
PCOS: Polycystic ovary syndrome
FD: Follicle density
